# Alteration of a Cry1A Shared Binding Site in a Cry1Ab-Selected Colony of *Ostrinia furnacalis*

**DOI:** 10.3390/toxins14010032

**Published:** 2022-01-02

**Authors:** Daniel Pinos, Yueqin Wang, Patricia Hernández-Martínez, Kanglai He, Juan Ferré

**Affiliations:** 1 Instituto de Biotecnología y Biomedicina (BIOTECMED), Deparment of Genetics, Universitat de València, 46100 Burjassot, Spain; daniel.pinos@uv.es (D.P.); patricia.hernandez@uv.es (P.H.-M.); 2 The State Key Laboratory for Biology of Plant Diseas and Insect Pests, Institute of Plant Protection, Chinese Academy of Agricultural Sciences, Beijing 100193, China; wangyueqin@caas.cn (Y.W.); hekanglai@caas.cn (K.H.)

**Keywords:** Asian corn borer, *Bacillus thuringiensis*, Cry1 toxins, binding site model, pyramid strategy

## Abstract

The Asian corn borer, *Ostrinia furnacalis* (Guenée, 1854), is a highly damaging pest in Asia and the Pacific islands, and larvae feed mainly from corn crops. To determine the suitability of Bt-corn technology for the future control of this pest, understanding the potential to develop resistance to Cry1Ab and the basis of cross-resistance to other Cry1 proteins is of great interest. Here, we have explored the binding of Cry1A proteins to brush border membrane vesicles from two *O. furnacalis* colonies, one susceptible (ACB-BtS) and one laboratory-selected with Cry1Ab (ACB-AbR). The insects developed resistance to Cry1Ab and showed cross-resistance to Cry1Aa, Cry1Ac, and Cry1F. Binding assays with radiolabeled Cry1Ab and brush border membrane vesicles from susceptible insects showed that Cry1A proteins shared binding sites, though the results were not conclusive for Cry1F. The results were confirmed using radiolabeled Cry1Aa. The resistant insects showed a reduction of the specific binding of both Cry1Ab and Cry1Aa, suggesting that part of the binding sites were lost or altered. Competition binding assays showed full competition between Cry1Ab and Cry1Aa proteins in the susceptible colony but only partial competition in resistant insects, confirming the alteration of some, but not all, binding sites for these two proteins. The binding site model for Cry1A proteins in *O. furnacalis* is in agreement with the occurrence of multiple membrane receptors for these proteins.

## 1. Introduction

*Bacillus thuringiensis* (Bt) is known to produce many insecticidal proteins that, either as Bt-based pesticides or expressed in genetically modified crops, can effectively control different insect pests [1]. One highly effective tool to control stem borers is Bt corn, which co-expresses different Bt proteins, mainly from the Cry1 family [2]. The adoption of Bt corn expressing Cry1Ab has quickly expanded globally since it has been demonstrated to control the European corn borer, *Ostrinia nubilalis*, and other pests [3]. Bt corn is also considered a promising technology to control the Asian corn borer, *Ostrinia furnacalis*, a highly damaging insect that affects mainly this crop [4,5].

The mode of action of Bt insecticidal proteins involves, among other steps, binding to membrane molecules in the midgut (referred to as “receptors”). This step is not only responsible for the specificity of the toxic action, but it is also the main step responsible for developing high levels of resistance by alteration of the membrane receptor [6]. Competition binding studies, in which the inhibition of binding of a labeled protein by different proteins is determined, have provided models showing whether Bt proteins bind to more than one site and whether different proteins share binding sites. These binding site models have been useful to predict and understand the basis of cross-resistance to Bt proteins since the alteration of a shared binding site can confer resistance to more than one Bt protein [7,8]. This approach, along with the use of laboratory-selected resistant colonies, allows us to be one step ahead of the onset of resistance in the field, understanding possible mechanisms of resistance before they even take place. In the case of its sibling species *O. nubilalis*, the binding site model proposes the presence of three shared binding sites for Cry1Ab and Cry1Ac. In that model, the Cry1F protein could bind to two of the binding sites, and Cry1Aa could only bind to one [9]. Although a study performed with ligand blots revealed certain similarities in binding patterns between *O. nubilalis* and *O. furnacalis* [10], a binding site model for Cry1A proteins in *O. furnacalis* is still needed. The characterization of the binding sites of this pest, along with the analysis of cross-resistance patterns in a laboratory-selected resistant colony to the Cry proteins expressed in Bt crops, can provide valuable knowledge to decide which of the available alternatives is the best to efficiently control this pest.

In the present study, we have analyzed the binding properties, as well as the insecticidal potency, of Cry1A proteins and Cry1F in two *O. furnacalis* colonies, one susceptible (ACB-BtS) and one laboratory-selected colony resistant to Cry1Ab (ACB-AbR). The ACB-AbR colony developed cross-resistance to Cry1Aa, Cry1Ac, and Cry1F. Moreover, the results have shown that Cry1A proteins share binding sites in this insect species and that at least one of these sites has been altered in the resistant insects.

## 2. Results

### 2.1. Susceptibility of O. furnacalis Colonies ACB-BtS and ACB-AbR to Cry1 Proteins

There were significant differences in susceptibility to Cry1Aa, Cry1Ab, Cry1Ac and Cry1F between the ACB-BtS colony and the ACB-AbR colony (Table 1). The ACB-BtS colony was highly susceptible to all tested Cry1 proteins with LC_50_ values lower than 1 μg/g in all cases. Based on the LC_50_ values, the ACB-AbR colony evolved a >714-fold resistance to Cry1Ab compared to the ACB-BtS colony. Regarding the other Cry proteins, the ACB-AbR colony showed high levels of cross-resistance: 178-fold to Cry1Aa, and >192-fold to both Cry1Ac and Cry1F.

### 2.2. Binding of ^125^I-labeled Cry1Ab to Brush Border Membrane Vesicles (BBMV)

**Figure 1:**
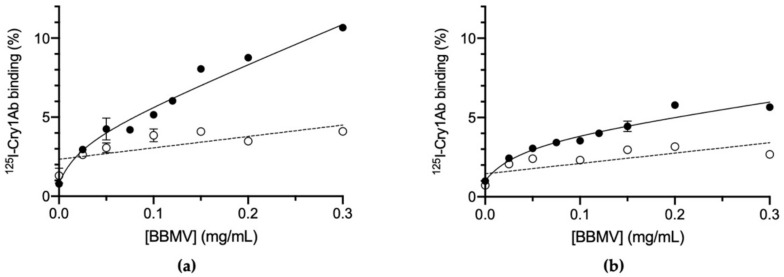
Binding of ^125^I-Cry1Ab at increasing concentrations of BBMV proteins. (**a**) *Ostrinia furnacalis* susceptible colony (ACB-BtS). (**b**) *O. furnacalis* Cry1Ab-resistant colony (ACB-AbR). Full dots represent total binding and empty dots represent non-specific binding. Each binding experiment was replicated at least twice and the error bars represent the standard error of the mean.

The Cry1Ab protein showed specific binding to BBMV from both susceptible and resistant colonies (Figure 1). At the maximum concentration of BBMV tested, a total of 10.7% of the labeled protein bound to BBMV from the ACB-BtS colony, whereas only a total of 5.5% of the labeled protein bound with BBMV from the ACB-AbR colony. In both samples, the non-specific binding at the maximum concentration of BBMV tested was ca. 3%. The resistant insects apparently lost approximately half the specific binding to Cry1Ab compared to the susceptible insects. The dissociation constants (K_d_) obtained from the homologous competition assays were 7.75 (±1.97) nM and 2.40 (±0.98) nM (mean ± SEM), and the concentration of binding sites (R_t_) were 1.95 (±0.28) and 0.60 (±0.16) pmol/mg (mean ± SEM) for the susceptible and resistant insects, respectively. The concentration of receptors (R_t_) for Cry1Ab is decreased by almost three-fold in the resistant colony, compared to the susceptible colony.

**Figure 2:**
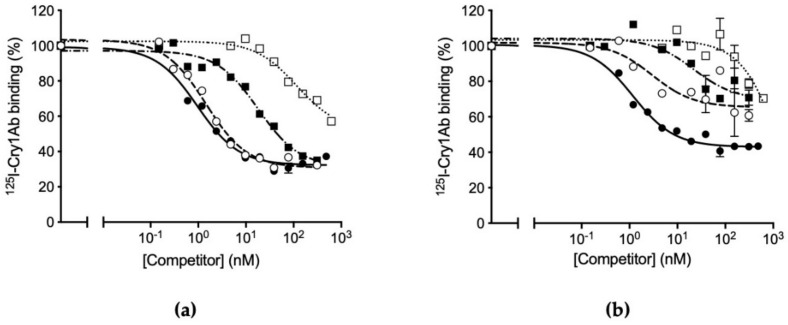
Competition binding assays with ^125^I-Cry1Ab. (**a**) With BBMV from *Ostrinia furnacalis* susceptible colony (ACB-BtS) and (**b**) with BBMV from *O. furnacalis* Cry1Ab-resistant colony (ACB-AbR). Curves represent total binding of ^125^I-Cry1Ab at increasing concentrations of unlabeled competitor: Cry1Aa (empty circles), Cry1Ab (full circles), Cry1Ac (full squares), or Cry1F (empty squares). Each competition experiment was replicated three times and the error bars represent the standard error of the mean.

The results from competition binding assays between labeled Cry1Ab and the other proteins are shown in Figure 2. For the susceptible colony (Figure 2a), the competition curve of Cry1Aa is very similar to that of the homologous competitor (Cry1Ab), indicating that Cry1Aa can bind to the same sites and with a similar affinity as Cry1Ab. For Cry1Ac, the competition curve indicates that, although it can displace all Cry1Ab binding sites, a higher concentration is required indicating a lower affinity for the Cry1Ab sites. For Cry1F, the curve indicates a much lower affinity for Cry1Ab binding sites and it is not clear if it would compete for all sites at higher concentrations. In the case of the resistant colony, both Cry1Aa and Cry1Ac are unable to compete for all Cry1Ab binding sites, strongly suggesting that one of the binding sites, shared by the three Cry1A proteins, has been altered, preventing Cry1Aa and Cry1Ac binding, though still allowing Cry1Ab to bind (Figure 2b). However, since all the heterologous proteins are still able to compete partially with Cry1Ab, other shared binding sites must remain. Since competition of Cry1F occurs at such a high concentration, it is not possible to draw any conclusion whether the alteration of the shared binding site affects this protein.

### 2.3. Binding of ^125^I-labeled Cry1Aa to BBMV

**Figure 3:**
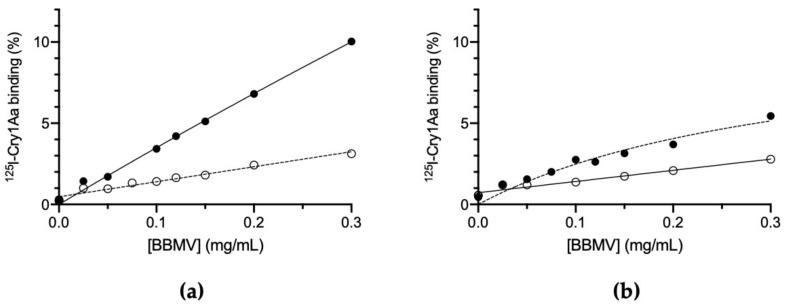
Binding of ^125^I-Cry1Aa at increasing concentrations of BBMV proteins. (**a**) *Ostrinia furnacalis* susceptible colony (ACB-BtS). (**b**) *O. furnacalis* Cry1Ab-resistant colony (ACB-AbR). Full dots represent total binding and empty dots represent non-specific binding. Each binding experiment was replicated at least twice and the error bars represent the standard error of the mean.

Since the competition curves using labeled Cry1Ab with the susceptible BBMV showed a similar behavior of Cry1Ab and Cry1Aa unlabeled competitors, to validate the binding site model we further labeled Cry1Aa. To directly determine whether its binding was also affected in the resistant insects, we tested labeled Cry1Aa with BBMV of both colonies. Figure 3 shows that both colonies showed specific binding of Cry1Aa to BBMV, with a clear reduction in the resistant colony compared to the susceptible one. The K_d_ values obtained were 0.48 (±0.03) and 0.79 (±0.24), and the R_t_ values were 0.22 (±0.02) and 0.13 (±0.02), for the susceptible and resistant colonies, respectively. The concentration of receptors (R_t_) for Cry1Aa is decreased by almost half in the resistant colony, compared to the susceptible colony.

**Figure 4:**
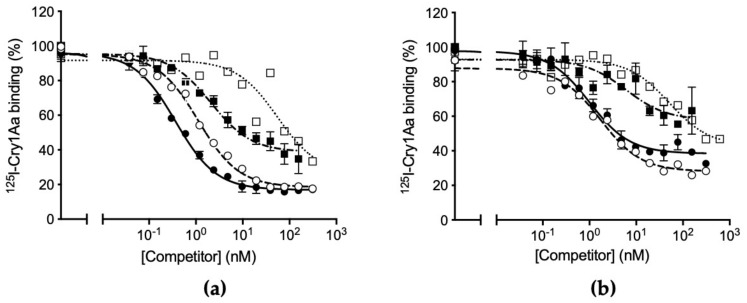
Competition binding assays with ^125^I-Cry1Aa. (**a**) With BBMV from *Ostrinia furnacalis* susceptible colony (ACB-BtS) and (**b**) with BBMV from *O. furnacalis* Cry1Ab-resistant colony (ACB-AbR). Curves represent total binding of ^125^I-Cry1Aa at increasing concentrations of unlabeled competitor: Cry1Aa (empty circles), Cry1Ab (full circles), Cry1Ac (full squares), or Cry1F (empty squares). Each competition experiment was replicated three times and the error bars represent the standard error of the mean.

Competition binding assays between labeled Cry1Aa and the other proteins are shown in Figure 4. Again, for the susceptible colony, both Cry1Aa and Cry1Ab compete with similar affinity for all sites occupied by labeled Cry1Aa (Figure 4a). In contrast, Cry1Ac could only compete for part of the Cry1Aa binding sites. The Cry1F protein shows a low ability to displace the labeled Cry1Aa binding, indicating a low affinity for certain Cry1Aa sites. For the resistant colony, although all heterologous proteins compete for Cry1Aa binding, both Cry1Ab and Cry1Ac cannot compete for all Cry1Aa binding, reinforcing the idea that at least one common binding site to Cry1A proteins is lost in the ACB-AbR colony (Figure 4b).

## 3. Discussion

The bioassays of the Cry1Ab-resistant colony (ACB-AbR) showed a resistance ratio for Cry1Ab of >714-fold, and cross-resistant levels of >192-fold for Cry1Ac and Cry1F. In addition, we also assessed the cross-resistance to Cry1Aa, which was 178-fold. In earlier characterizations of the resistant colony, where the Cry1Ab resistance ratio reached 40-fold, an initial cross-resistance of 37-fold to Cry1Ac was observed [11], which increased later to 113-fold [12], indicating that the selection for resistance to Cry1Ab produced a remarkable cross-resistance to Cry1Ac in the ACB-AbR colony. Interestingly, for the Cry1F protein, an initial low cross-resistance of six-fold was detected in the ACB-AbR colony [11], which increased to a notable 48-fold resistance in the latter study [12] achieving very high levels in the present study.

**Figure 5:**
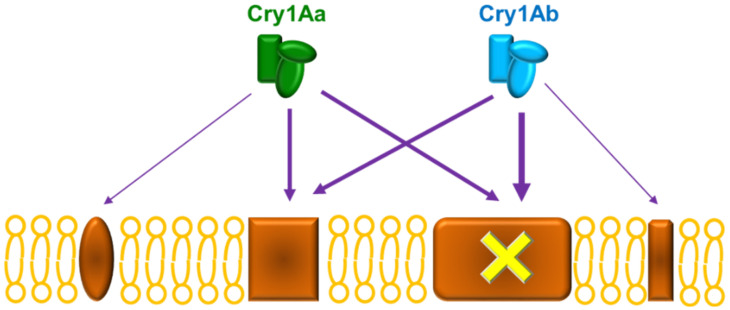
Binding site model for Cry1Aa and Cry1Ab proteins in *Ostrinia furnacalis*. The yellow cross represents the altered binding site in the ACB-AbR colony. The width of the arrows represents the relative relevance of the binding sites. Cry1Aa and Cry1Ab may have, in addition to the shared sites, other sites with a minor contribution to the toxicity and which can be shared with other Cry proteins.

The use of competition binding experiments can provide binding site models that help us predict or understand the biochemical basis of patterns of cross-resistance in a resistant colony [7,8]. In *O. furnacalis*, our results from competition binding assays suggest that there are at least two major binding sites for Cry1Aa and Cry1Ab, which are shared by these two proteins (Figure 5). According to the competition curves, Cry1Ac competes for all Cry1Ab binding sites (Figure 2a), whereas it cannot compete for all Cry1Aa binding sites (Figure 4a), suggesting the occurrence of additional binding sites for this protein. The heterologous competition of Cry1F only at very high concentration may indicate that, though it can recognize with low affinity some of the Cry1A receptors, it must have singular receptors to exert its toxic action. This binding site model shares certain similarities with the model reported for the phylogenetically close species *Ostrinia nubilalis*, in which Cry1Ab and Cry1Ac competed for all binding sites and Cry1F only competed at higher concentrations [8,9]. The competition of Cry1F for the binding sites of Cry1A proteins has been shown to be of low affinity in many insect pests, such as *Plutella xylostella*, *Heliothis virescens*, *Helicoverpa armigera*, and *Helicoverpa zea* [13,14,15].

For the resistant colony, the binding results indicate that Cry1Ab and Cry1Aa have lost part of their binding capacity, presumably by alteration of one of its shared binding sites (Figure 1 and Figure 3). The fact that the heterologous competitors can only displace part of the ^125^I-Cry1Ab and ^125^I-Cry1Aa binding in the resistant insects (Figure 2b and Figure 4b) is indicative of the occurrence of, at least, two types of binding sites, one that will be shared with the heterologous competitors (the one that they are able to compete) and another one that is not shared (the part of the labeled protein that cannot be displaced). Taking into account the results with the susceptible insects, in which Cry1Aa and Cry1Ac could completely displace binding of ^125^I-Cry1Ab (Figure 2a), the most plausible explanation is that one of the two Cry1Ab binding sites has suffered an alteration in the resistant insects that, though still allowing binding of Cry1Ab, prevents binding of the other Cry proteins (Figure 3).

It is well known that Cry1 proteins use several membrane proteins as receptors in the midgut of lepidopteran larvae, such as aminopeptidase N, cadherin, and ABC transporters [16,17,18,19]. In *O. furnacalis*, a cadherin was first found altered in a Cry1Ac-resistant colony (ACB-AcR) [20]. A later study characterized the alteration of aminopeptidase N and ABC type G transcripts in both the ACB-AbR colony (studied here) and the ACB-AcR colony [21]. Recently, the involvement of the *O. furnacalis* cadherin in the toxicity of Cry1Aa and Cry1Ac was proven through CRISPR knock-outs [22], as well as the ABCC2 in the toxicity of Cry1F and Cry1Ab/c [23]. The binding sites here proposed could be located in some of these receptors or even in different epitopes of the same one, as has been shown for Cry1A proteins and the ABCC2 transporter in *Spodoptera exigua* [24,25]. Previous studies have linked the resistance to Cry1Ac to the modulation of the expression of many midgut genes by *trans-*regulatory signaling mechanisms in *Plutella xylostella* [26,27]. In our case, this possibility is plausible since the inheritance of the resistance in the ACB-AbR colony is polygenic [12].

According to our binding site model, *O. furnacalis* has, at least, two major shared binding sites for Cry1A proteins. As a consequence, the alteration of one of the binding sites may contribute to the cross-resistance observed among Cry1A proteins. The cross-resistance to Cry1F seems not to be due to the alteration of Cry1A binding sites, thus other selected mechanisms may be responsible. Binding site models, along with cross-resistance data from laboratory-selected resistant colonies, are important tools that can help decision-making for effective pyramiding of Bt-crops to combat resistant evolution in insect pest populations.

## 4. Materials and Methods

### 4.1. Insect Colonies

The *O. furnacalis* Bt susceptible colony (ACB-BtS) was originally collected from Huxian, Shaan’xi province (China), and maintained in laboratory conditions using a semi-artificial diet as previously described [28]. The *O. furnacalis* Cry1Ab resistant colony (ACB-AbR) was selected from a sample of the Bt susceptible colony by exposure to trypsin-activated Cry1Ab. After an initial exposure (2.5 ng of Cry1Ab/g diet), the protein concentration was increased to target 40–70% mortality. After 51 generations, larvae were reared on diet containing 400 ng of toxin/g diet. This colony achieved a >40-fold resistance to the protoxin form of Cry1Ab after 71 generations [11]. From generation 124, the protein concentration was increased to 2.0 μg/g. Thereafter, the colony has been maintained at this concentration. Larvae used for dissecting midguts in this study were from generation 208. Bioassays were carried out at generation 211 to determine the LC_50_s of Cry1Aa, Cry1Ab, Cry1Ac, and Cry1F.

### 4.2. Bt Proteins Preparation

*Bacillus thuringiensis* Cry proteins were obtained from recombinant strains EG1273, EG7077, EG11070, EG11069 expressing Cry1Aa, Cry1Ab, Cry1Ac, and Cry1Fa, respectively (from Ecogen Inc., Langhorn, PA). For bioassays, Cry proteins were purified and solubilized as previously described [29], stored lyophilized, and resuspended in the appropriate buffer before use. For binding assays, Cry proteins were activated by trypsin and then dialyzed in 20 mM Tris-HCl (pH 9) and filtered. Then, they were purified by anion-exchange chromatography in a HiTrap Q HP column using an ÄKTA explorer 100 chromatography system (GE Healthcare, Little Chalfont, United Kingdom). The Cry1Ab protein used for iodine labeling was further purified by size-exclusion chromatography with a Superdex 200 column (GE Healthcare, Little Chalfont, United Kingdom) using the same system. The purity of all proteins was analyzed by 12% sodium dodecylsulfate polyacrylamide gel electrophoresis (SDS-PAGE) (SE 250 Minivertical Unit, Cytiva, Marlborough, MA, USA). All proteins were kept at −20 °C until used.

### 4.3. Diet Bioassays

Larval susceptibility of *O. furnacalis* susceptible and resistant insects were evaluated by diet incorporation assays in an agar-free semi-artificial diet, as previously described [30]. Briefly, neonates were individualized in 48-well trays containing the diet with different concentrations of proteins. Trays were held at 27 ± 1 °C, 80% RH, and a 16:8 h photoperiod. Survivors and their weights were recorded after seven days. When the mortality was calculated, the larvae were considered dead if they died (visually motionless while poked with a fine brush) or weighed ≤0.1 mg. Bioassay data were subjected to Probit analysis with PoloPlus (PoLo, Version 1.0, LeOra Software, Petaluma, CA, USA) to obtain the LC_50_ values for each protein [31].

### 4.4. BBMV Preparation

BBMV were prepared from dissected midguts obtained from fifth instar larvae of both ACB-BtS and ACB-AbR colonies by the differential magnesium precipitation method [32] and kept at −80 °C until used. The protein concentration of BBMV preparations was determined by the method of Bradford [33].

### 4.5. Binding Assays with ^125^I-Labeled Cry1Ab and Cry1Aa

The trypsin-activated and chromatography purified Cry1Ab and Cry1Aa proteins were labeled using the chloramine-T method [34]. Twenty-five micrograms were mixed with 0.3 mCi of ^125^I (Perkin Elmer, Boston, MA, USA), and 6 mM of chloramine-T for 45 s at room temperature (RT). After incubation, the reaction was stopped by adding sodium metabisulfite (5.75 mM) followed by sodium iodide (250 mM). The specific activity obtained for the labeled Cry1Ab was 1.1 mCi/mg and 3.8 mCi/mg for Cry1Aa.

Prior to use, BBMV from susceptible and resistant colonies were centrifuged for 10 min at 16,000× *g* and resuspended in binding buffer (phosphate-buffered saline, (PBS), 0.1% BSA). To determine the optimal concentration of BBMV for use in competition experiments, increasing amounts of BBMV were incubated with 1.22 nM of labeled Cry1Ab, in a final volume of 0.1 mL of binding buffer for 1 h at RT. An excess of the same unlabeled protein (>2000 nM) was used to estimate the non-specific binding. The specific binding was calculated as the subtraction of the total binding minus the non-specific binding. Homologous (using the same unlabeled protein as a competitor) and heterologous (using other proteins as competitors) competition experiments were performed in binding buffer incubating 0.2 mg/mL of BBMV with ^125^I-labeled proteins and increasing amounts of unlabeled proteins in a final volume of 0.1 mL for 1 h at RT. After incubation, samples were centrifuged at 16,000× *g* for 10 min. Then, pellets were washed with 0.5 mL of binding buffer and centrifuged again. Radioactivity in the pellets was measured in a model 2480 WIZARD^2^ gamma counter (Perkin Elmer, Boston, MA, USA). The equilibrium dissociation constant (*K*_d_) and concentration of binding sites (*R*_t_) were estimated from the homologous competition experiments for each colony using the LIGAND program [35].

## Figures and Tables

**Table 1:** Toxicity of *Bacillus thuringiensis* proteins to neonate larvae of *Ostrinia furnacalis* after 7 days of exposure.

Protein Tested	Colony	N ^a^	LC_50_ (95% FL) ^b^ (μg protein/g Diet)	Slope (±SE)	χ^2^	df (χ^2^)	RR ^c^ (95% CI)
Cry1Ab	ACB-BtS	768	0.28 (0.20–0.36)	1.55 ± 0.13	5.2	14	-
	ACB-AbR	864	>200 ^d^	-	-	-	>714
Cry1Aa	ACB-BtS	768	0.18 (0.15–0.22)	1.90 ± 0.13	8.6	14	-
	ACB-AbR	864	32 (25–40)	1.62 ± 0.15	7.6	-	178 (131–237)
Cry1Ac	ACB-BtS	768	0.26 (0.19–0.34)	1.36 ± 0.11	9.5	16	-
	ACB-AbR	768	>50 ^d^	-	-	-	>192
Cry1F	ACB-BtS	864	0.52 (0.36–0.70)	1.33 ± 0.11	6.4	14	-
	ACB-AbR	672	>100 ^d^	-	-	16	>192

^a^ Number of larvae tested in bioassays. ^b^ Concentration of protein killing 50% of larvae and its 95% fiducial limits. ^c^ Resistance ratio and its 95% confidence interval compared with the susceptible colony at LC_50_. ^d^ Mortalities [(mean ± SD) %] were 39.6 ± 2.1, 40.6 ± 1.0 and 35.4 ± 2.1 at the highest concentrations of Cry1Ab (200 μg/g), Cry1Ac (50 μg/g) and Cry1F (100 μg/g), respectively. Due to the limitation in the amount of protein available, we were unable to increase the concentrations.
